# Role of heat shock protein 70 in silibinin-induced apoptosis in bladder cancer

**DOI:** 10.7150/jca.88668

**Published:** 2024-01-01

**Authors:** Yi Wei, Yanxin Zhuang, Yishuai Zhang, Lei Luo, Bixin Yu, Jin Zeng

**Affiliations:** 1 Department of Urology, The First Affiliated Hospital of Xi'an Jiaotong University, Xi'an, Shaanxi 710061, P.R. China.; 2 Department of Ophthalmology, The First Affiliated Hospital of Xi'an Jiaotong University, Xi'an, Shaanxi 710061, P.R. China.; 3 Department of Medical Oncology, The First Affiliated Hospital of Xi'an Jiaotong University, Xi'an, Shaanxi 710061, P.R. China.

**Keywords:** apoptosis, bladder cancer, HSF1, Hsp70, silibinin

## Abstract

Hsp70 (heat shock protein 70) plays critical roles in cancer cell proliferation and apoptosis. Recently, accumulating evidences have demonstrated the cancer promoting effects of Hsp70 in bladder cancer. The development of novel therapeutic approaches targeting Hsp70 thus received great attention from researchers. In this study, we demonstrated that silibinin, a natural polyphenolic flavonoid isolated from the milk thistle, targeted Hsp70 by inhibiting its transcription in bladder cancer cells. We also demonstrated that knockdown of endogenous Hsp70 enhanced silibinin-induced apoptosis, while overexpression of exogenous Hsp70 could partially reverse the effects of silibinin-induced cell apoptosis. Furthermore, we found that silibinin could activate HSF1/Hsp70-regulated mitochondrial apoptotic pathway. Mechanically, silibinin inhibited the interaction between Apaf-1 and Hsp70, thus increasing the recruitment of pro caspase-9. Results from *in vivo* study demonstrated that silibinin suppressed the growth of bladder cancer xenografts, which was accompanied with the activation of caspase-3 and downregulation of HSF1 and Hsp70. Taken together, our data indicates that silibinin induces mitochondrial apoptosis *via* inhibiting HSF1/Hsp70 pathway and also suggests the therapeutic potential of silibinin in the treatment of bladder cancer.

## Introduction

Bladder cancer is one of the most common malignancies worldwide. Superficial bladder cancer accounts for approximately 70% of all bladder cancer cases^1^. The standard treatment for patients with superficial disease is transurethral resection (TUR) of tumors. Post-TUR intravesical chemotherapy/immunotherapy is widely used as an adjuvant therapy to prevent recurrence and progression of superficial bladder cancer after TUR^2^, ^3^. However, the current therapy fails to exert significant anti-tumor effect due to a defective apoptotic machinery or resistance of the bladder cancer cells to the specific death mechanisms. Thus, new and alternative agents need to be explored.

There have been numerous studies directed toward the identification of natural agents for the prevention and intervention of bladder cancer during the past few years. Silibinin is one of the most popular dietary supplements and has been extensively studied for its anti-oxidant, hepatoprotective and anti-cancer properties^4^-^6^. Accumulating evidence has indicated the cancer-preventive and therapeutic effects of silibinin in different animal tumor models and cancer cells, including cancers of prostate, breast, colon, lung, kidney, and bladder^7^-^12^. Our lab has been focusing on the molecular mechanisms of silibinin against urological cancers for decades. Recent research has shown that silibinin could inhibit epithelial-mesenchymal transition (EMT) of renal cancer through activating autophagy-dependent β-catenin degradation^13^. Moreover, we have latterly indicated that silibinin could attenuate metastasis and suppress EMT in bladder cancer via downregulating COX-2^14^. Since the broad-spectrum anti-tumor activity of silibinin has been aware by researchers, there is an increasing interest in identifying the new mechanisms for silibinin on cancers.

Induction of apoptosis is believed to be one of the major mechanisms for silibinin against cancer cells, although the details are yet to be elucidated^15^, ^16^. Several reports suggest that silibinin induces apoptosis through caspases activation and downregulation of survivin in human bladder carcinoma cells^17^, ^18^. Additionally, oral administration of silibinin has been reported to prevent N-butyl-N-(4-hydroxybutyl) nitrosamine (OH-BBN)-induced mouse bladder carcinogenesis^19^. The study conducted by our group has also shown that silibinin induced apoptosis in human bladder cancer 5637 cells *in vitro* and *in vivo*, which was associated with the activation of cytochrome c (Cyto C)/caspase-dependent and AIF/caspase-independent pathways involving selective release of Omi/HtrA2 from mitochondria^17^. However, the exact mechanism responsible for the apoptotic effect of silibinin is not thoroughly understood yet.

In the recent years, heat shock proteins (HSPs) have been identified as key determinants of cancer cell survival, which can modulate apoptosis by directly interacting with components of the apoptotic machinery^20^, ^21^. Manipulation of the HSPs by chemical agents could represent a viable strategy against cancer cells. Therefore, the purpose of this study was to examine the possible role of HSPs in regulating silibinin-induced apoptosis in bladder cancer cells. In the present study, using *in vitro* and *in vivo* bladder cancer models, we explored the molecular mechanisms of silibinin-induced apoptosis, focusing on Hsp70-regulated mitochondrial apoptotic pathway.

## Materials and Methods

### Cell Culture and Heat Treatment

The human bladder transitional cell carcinoma RT4, 5637, T24 cell lines were obtained from American Type Culture Collection (ATCC). Cells were cultured in RPMI-1640 (5637 cell line) or DMEM (RT4 and T24 cell lines) containing 10% fetal bovine serum (FBS, Gibco, Thermo Fisher Scientific, Inc.) and 1% penicillin-streptomycin (Gibco; Thermo Fisher Scientific, Inc.) at 37 °C.

Heat treatment was applied by incubating the T24 cells at 42 °C for 24 hours. Then the cells were treated with DMSO or silibinin (200 μM) for another 24 hours at 37 °C followed by flow cytometry analysis or western blotting.

### Reagents

Silibinin, MTT (3-[4, 5-dimethylthiazol-2-yl]-2, 5-diphenyltetrazolium bromide) and hydrogen peroxide (H_2_O_2_) were purchased from Sigma‑Aldrich; Merck KGaA. We treated the cells with 200 μM H_2_O_2_ at 37 °C for 24 hours to simulate the oxidative stress. Antibodies against Histone H1 (cat. no. sc-393358; 1:1000), glyceraldehyde-3-phosphate dehydrogenase (GAPDH) (cat. no. sc-47724; 1:1000) and cytochrome c (cat. no. sc-13156; 1:1000) were from Santa Cruz Biotechnology, Inc. (Santa Cruz, CA). Antibodies against cleaved caspase-9 (cat. no. 9505; 1:1000), cleaved caspase-3 (cat. no. 9664; 1:1000), cleaved caspase-8 (cat. no. 9748; 1:1000), poly (ADP-ribose) polymerase (PARP) (cat. no. 9542; 1:1000), cytochrome c oxidase IV (COX-IV) (cat. no. 4844; 1:1000), Hsp27 (cat. no. 2402; 1:1000), Hsp70 (cat. no. 4872; 1:1000), Hsp40 (cat. no. 4868; 1:1000), Hsp60 (cat. no. 12165; 1:1000), Hsp90 (cat. no. 4875; 1:1000), and c-Raf (cat. no. 53745; 1:1000) were from Cell Signaling Technology, Inc. (Beverly, MA). The enhanced chemiluminescence (ECL) detection system (cat. no. 1705062) was from Bio‑Rad Laboratories, Inc. (Hercules, CA).

### Cell Viability Assay

Cell viability was assessed using MTT assay. Bladder cancer cells were incubated in the absence or presence of silibinin for various times, and incubated with 0.5 mg/ml MTT at 37 °C for 4 hours. After incubation, cells were lysed with dimethyl sulfoxide (DMSO). The absorbance was determined using a 96-well microplate reader (Bio-Rad, Hercules, CA) at wavelength of 570 nm. The experiments were performed in triplicate.

### Quantitative Detection of Apoptosis

After indicated treatment, cells were collected and subjected to annexin V and propidium iodide (PI) staining using an Annexin V-FITC Apoptosis Detection Kit (cat. no. 556547; BD Biosciences, San Jose, CA), following the protocol provided by the manufacturer. Apoptotic cells were then analyzed by flow cytometry (BD Biosciences, San Jose, CA).

### Preparation of Mitochondria, Cytosolic and Nuclear Fractions

Briefly, cells were homogenized in permeabilization buffer (250 mM sucrose, 20 mM HEPES/KOH [pH 7.4], 1 mM EGTA, 1 mM EDTA) containing complete protease inhibitor cocktail (Roche Diagnostics Ltd, Mannheim, Germany), and centrifuged at 500 g (4 °C) for 10 min to pelletize the nucleus and cell debris. The nuclear pellets were suspended in RIPA lysis buffer (100 mM Tris-HCl [pH 7.4], 150 mM NaCl, 1 mM EDTA, 1% Triton X-100, 1% sodium deoxycholate, 0.1% SDS) containing complete protease inhibitor cocktail (Roche Diagnostics Ltd, Mannheim, Germany). After vortex and centrifugation at 15,000 g (4 °C) for 15 min, the nuclear supernatants were collected. The supernatants were further centrifuged at 15,000 g for 30 min. Cytosolic supernatants and crude mitochondrial pellets were collected. The pellets were then suspended in mitochondrial lysis buffer (150 mM NaCl, 50 mM Tris-HCl [pH 7.4], 1% NP-40, 0.25% sodium deoxycholate, 1 mM EGTA) containing complete protease inhibitor cocktail (Roche Diagnostics Ltd, Mannheim, Germany). After vigorous vortex and centrifugation at 15,000 g (4 °C) for 15 min, the soluble mitochondrial supernatants were collected.

### Western Blotting

Cell lysates were prepared in RIPA lysis buffer (100 mM Tris-HCl [pH 7.4], 150 mM NaCl, 1 mM EDTA, 1% Triton X-100, 1% sodium deoxycholate, 0.1% SDS) containing complete protease inhibitor cocktail (Roche Diagnostics Ltd, Mannheim, Germany). For immunoblot analyses, 30-60 μg samples of protein were subjected to SDS-PAGE on 10% or 15% Tris-glycine gels and separated proteins were transferred onto PVDF membranes. Membranes were blocked with 5% milk for 1 hour at room temperature and probed with primary antibodies against desired molecules over night at 4 °C, following by the anti-rabbit IgG peroxidase antibody produced in goat (cat. no. A9169; 1:5,000; Sigma‑Aldrich; Merck KGaA) or anti-mouse IgG peroxidase antibody produced in goat (cat. no. A4416; 1:5,000; Sigma‑Aldrich; Merck KGaA) for 1 hour at room temperature. The bands of proteins were visualized using the ECL detection system followed by exposure to X-ray film. The relative intensity of each band was determined by using Glyko BandScan software (Glyko, Novato, USA).

### Dual-Luciferase Assay

HSF1 firefly luciferase reporter plasmid (pGL4.41 [luc2P/HSE/Hygro] vector) and the pRL (Renilla) luciferase reporter plasmid (pRL-TK) were purchased from Promega. T24 bladder cancer cells were co-transfected with 100 ng HSF1 reporter plasmid and 200 ng pRL-TK plasmid using Lipofectamine 2000 (Invitrogen, Carlsbad, CA, USA). 24 hours after transfection, cells were treated with indicated concentration of silibinin for another 24 hours. The firefly luciferase and Renilla luciferase activities were measured using a Dual-Luciferase Reporter Assay System kit (cat. no. E1910, Promega) in accordance with the manufacturer's protocol. Relative light units (RLUs) were measured in 96-well microplate reader (Bio-Rad, Hercules, CA). Values were normalized to Renilla activity and relativized to the control group.

### Mitochondrial Membrane Potential (ΔΨm) Assay

In this study, ΔΨm was determined using JC-1 probes (cat. no. 551302; BD Bioscience, San Jose, CA). Cells were harvested and centrifuged at 400 g (4 °C) for 5 min. The cell pellet was resuspended in 0.5 ml of JC-1 solution and incubated at 37 °C for 20 min. After rinsing, cells were analyzed by flow cytometry (BD Biosciences, San Jose, CA). A dot plot of red fluorescence (FL2, living cells with intact ΔΨm) versus green fluorescence (FL1, cells with lost ΔΨm) was recorded. Cells with dissipated ΔΨm were then measured.

### siRNA and Transfection

Cells were transfected with small interference (si) RNA targeting Hsp70 or with control siRNA using Lipofectamine 2000 (Invitrogen, Carlsbad, CA) in accordance with the manufacturer's instructions. The corresponding negative control (siNC) 5'‑UUCUCCGAACGUGUCACGUTT‑3' was designed and synthesized by Guangzhou RiboBio Co., Ltd. The sequences of the siRNAs used in the present study were as follows: Hsp70 siRNA #1 sense, 5'-GCCUUUCCAAGAUUGCUGUUU-3'; Hsp70 siRNA #2 sense, 5'-CACUACCUUUUUCGAGAGU-3'; Hsp70 siRNA #3 sense, 5'-GAGCUUCAAGACUUUGCAU-3'. Cells were cultured for 48 hours after transfection and then harvested for further experiments.

### Total RNA Extraction and Real-time RT-PCR Analysis

Total RNA was extracted from cells using a Total RNA Extraction Kit obtained from Fastagen (Shanghai, China). cDNA was synthesized using a Reverse Transcription Reaction Kit (MBI Fermentas, St. Leon-Rot, Germany) and then amplified using specific primers. Primer sequences were listed as follows: Hsp70 (forward primer, 5'-CGGCAAGGTGGAGATCAT-3'; reverse primer, 5'-GGTGTTCTGCGGGTTCAG-3') and GAPDH (forward primer, 5'-GGAGCGAGATCCCTCCAAAAT-3'; reverse primer, 5'-GGCTGTTGTCATACTTCTCATGG-3'). The PCR reaction parameters were set follows: 5 min at 95 °C and 30 cycles of 30 s at 95 °C, 30 s at 58 °C and 45 s at 72 °C, followed by 10 min at 72 °C. Relative changes in gene expression were normalized against the internal control gene, GAPDH.

### Establishment of Stable Expression Cell Clones

The pEGFP Hsp70 plasmid (Addgene plasmid 15215) was transfected into T24 cells using Lipofectamine 2000 (Invitrogen, Carlsbad, CA, USA). After 48 hours, the tranfected cells were subcultured at the ratio of 1:3, then were subjected to G418 selection at the final concentration of 450 ng/ml. The transfected cells were continued to culture in the presence of G418 selection for 21 days to enable enrichment of cells expressing Hsp70. To confirm expression, transfected cells were harvested and the respective lysates were probed for the expression of Hsp70 *via* western blotting analysis.

### Co-immunoprecipitation

Immunoprecipitations (IP) were carried out using Immunoprecipitation kit, Dynabeads protein G (Invitrogen) following the manufacturer's instructions. Briefly, protein G Dynabeads were coated with rabbit anti-Apaf-1 (cat. no. sc-65891; Santa Cruz Biotechnology, CA) under rotation for 1 hour at 4 °C. In order to eliminate antibody contamination in precipitated proteins, Apaf-1 antibody was cross linked to Dynabeads protein G using the cross-linking agent BS3 (cat. no. PG82083; Thermo Fisher Scientific) following the manufacturer's instructions. Cell lysates were prepared in IP lysis buffer (20 mM Tris-HCl [pH 7.5], 150 mM NaCl, 1 mM EDTA, 1 mM EGTA, 1% Triton X-100 and complete protease inhibitor cocktail). Dynabeads coated with antigen-antibody complex were washed extensively with IP buffer. The proteins on Dynabeads were eluted by boiling at 95 °C for 5 min in SDS sample loading buffer and separated on a 10% SDS-PAGE for western blotting.

### Xenograft Animal Models

4-week-old Balb/c male nude mice (n=12, 15-20 g) were obtained from the Laboratory Animal Center of Xi'an Jiaotong University. All animal care and experiments were approved by the Institutional Animal Care and Use Committee of Xi'an Jiaotong University. The T24 cells were respectively resuspended with serum-free medium at the density of 2.0×10^7^/ml containing matrigel (Sigma-Aldrich; Merck KGaA). Total 4.0×10^6^ cells suspension (200 μl) was subcutaneously injected to the right flank of nude mice. When the tumor volume reached about 150 mm^3^ in size, the mice were separated to two groups: T24 control group (n=6) and T24 silibinin treatment group (100 mg/kg, n=6). All the mice were operated every 3 days and the tumor volume was calculated as follows: Volume (mm^3^)=0.5×(length)×(width)^2^. Tumors were harvested after 30 days, and the mice were sacrificed using carbon dioxide (CO_2_) with a CO_2_ displacement rate of 17.5% of chamber volume/min. The animals were exposed to CO_2_ until complete cessation of breathing was observed for 10 min. Tumors were embedded with paraffin and submitted to immunohistochemistry staining and western blotting assay.

### Immunohistochemistry (IHC) Assay

Tumors were fixed in 4% paraformaldehyde and embedded with paraffin. Slides were prepared from cutting sections of each 5 μm embedded block. In brief, the slides were deparaffinized, hydrated and blocked. Then, the slides were washed 3 times with 0.01 M PBS and blocked with FBS for 1 hour at room temperature. After that, the slides were incubated with primary antibody against cleaved caspase-3, Hsp70 and HSF1 at 4 °C overnight, and incubated with the appropriate secondary antibodies at room temperature for 1 hour. The slides were stained using diaminobenzidine (DAB) kit following the manufacturer's instructions and photographed under an inverted microscope (Olympus Optical Co., Ltd., Tokyo, Japan).

### Statistical Analysis

All statistical analyses were performed using SPSS 15.0 (SPSS Inc, Chicago, IL). Quantitative data are presented as mean ± SE and the differences among the control and various treatment groups were compared by one-way ANOVA, followed by Dunnett's *t* test for separate comparisons. When the comparison involved only two groups, the Student's *t* test was used. *P* < 0.05 was considered statistically significant.

## Results

### Silibinin inhibits proliferation and induces apoptosis in bladder cancer cells

**Figure 1:**
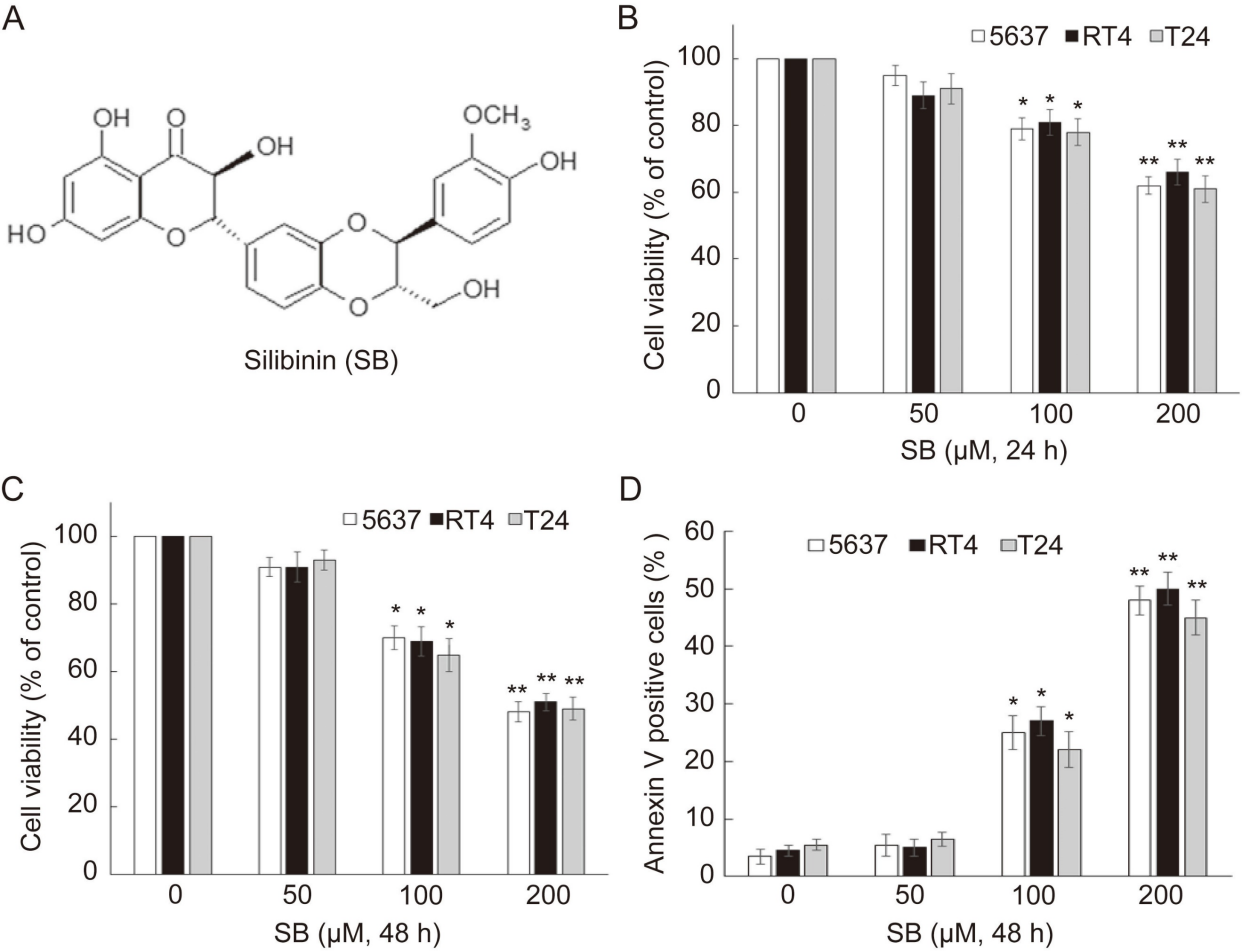
** Silibinin inhibits proliferation and induces apoptosis in bladder cancer cells.** (A) The chemical structure of silibinin. (B, C) Bladder cancer cell lines 5637, RT4 and T24 were treated with various concentrations of silibinin (0, 50, 100, 200 µM) for 24 hours or 48 hours. Cell viability was evaluated by an MTT assay. (D) Cells were incubated with different concentrations of silibinin for 48 hours, and then stained with Annexin-V-FITC/PI assay and analyzed by flow cytometry. *P<0.05, **P<0.01 vs control group. Results are shown as the mean ± standard deviation of three experimental repeats.

To evaluate the effects of silibinin on the growth of bladder cancer cells, 5637, RT4 and T24 were treated with different doses of silibinin (0, 50, 100, 200 µM) for 24 hours or 48 hours. Cell viability assay showed that silibinin could significantly inhibit proliferation of bladder cancer cells in a dose-dependent manner (Fig.1B and C). To determine whether silibinin could induce apoptosis, silibinin-treated bladder cancer cells were stained with Annexin V-FITC and PI. We observed that silibinin treatment increased the number of apoptotic cells in 5637, RT4 and T24 (Fig. 1D). The results indicate that silibinin suppresses proliferation and induces apoptosis in bladder cancer cells.

### Silibinin suppresses the expression of Hsp70 in bladder cancer cells

**Figure 2:**
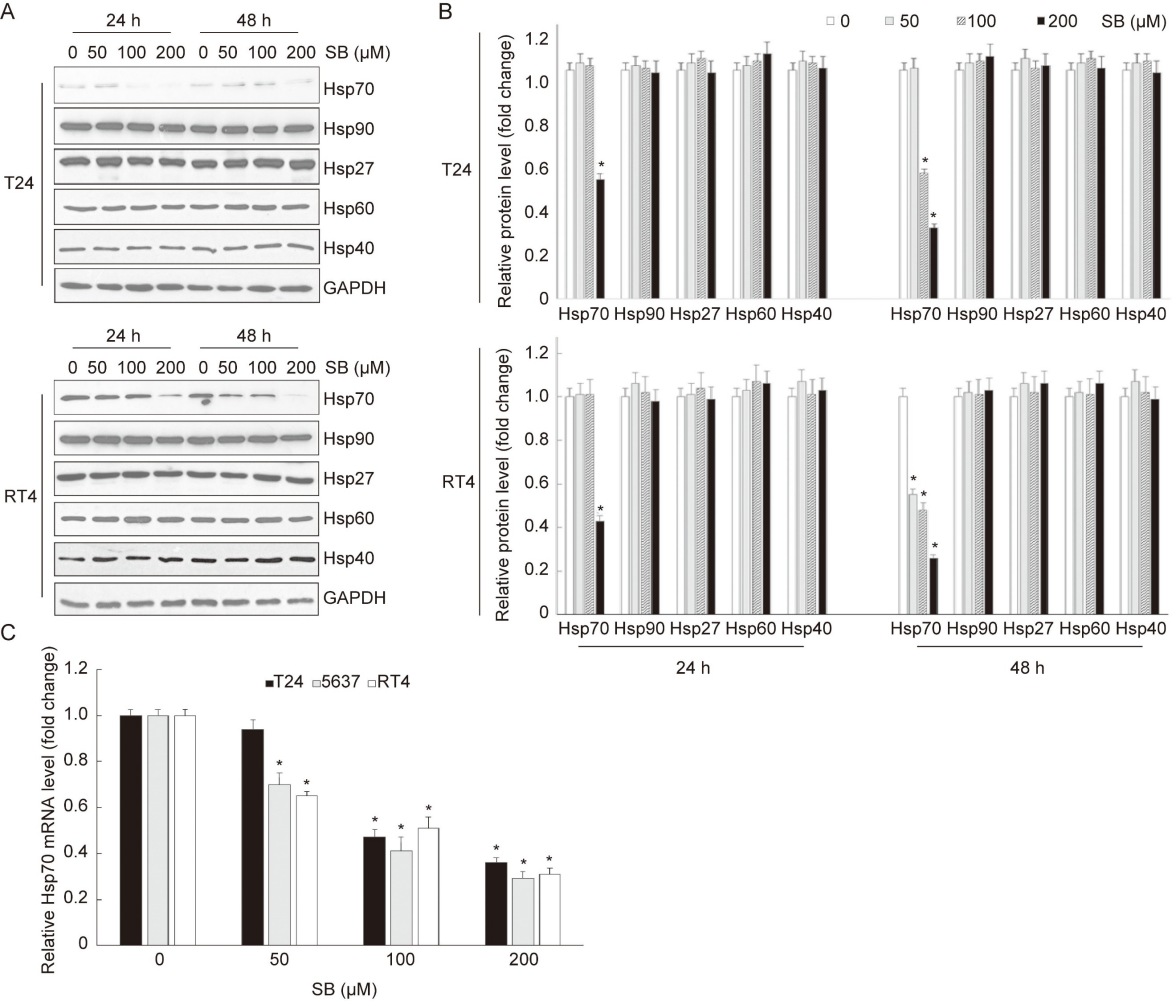
** Silibinin suppresses the expression of Hsp70 in bladder cancer cells.** (A) Bladder cancer cells T24 and RT4 were treated with silibinin (0, 50, 100 and 200 µM) for 24 hours or 48 hours, and the expressions of Hsp70, Hsp90, Hsp27, Hsp60, Hsp40 were detected by western blotting. (B) Relative expression levels of HSPs proteins. (C) T24, 5637 and RT4 cells were incubated with different concentrations of silibinin for 24 hours, the mRNA level of Hsp70 was detected by real-time PCR. *P<0.05 vs control group. Results are shown as the mean ± standard deviation of three experimental repeats.

Heat shock proteins (HSPs) are a large family of evolutionarily conserved proteins and can be divided into different groups according to their molecular weight^22^. It's widely accepted that HSPs play significant roles in the regulation of apoptosis^21^. In this study, to detect which HSPs was involved in silibinin-induced apoptosis, bladder cancer cells T24 and RT4 were treated with different doses of silibinin for 24 hours or 48 hours, respectively. Western blotting analysis showed that silibinin only suppressed Hsp70 in both T24 and RT4 cells (Fig. 2A and B). Furthermore, real-time PCR analysis showed that silibinin decreased Hsp70 mRNA level of T24, 5637 and RT4 cells in a dose-dependent manner (Fig. 2C). Taken together, our results show that silibinin suppresses the expression of Hsp70 in bladder cancer cells.

### Exogenous Hsp70 modulates silibinin-induced apoptosis in bladder cancer cells

**Figure 3:**
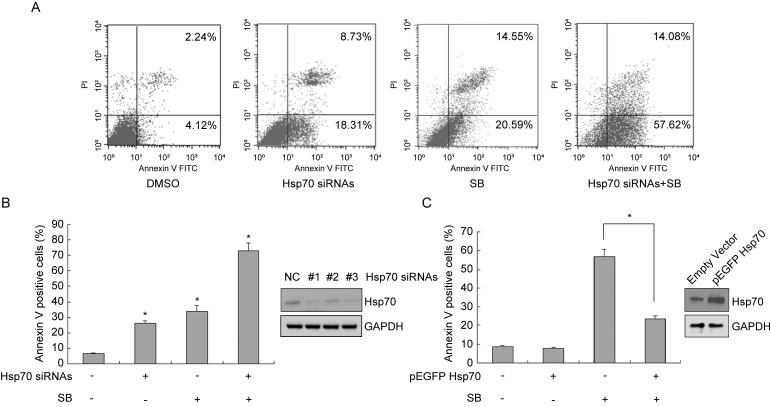
** Exogenous Hsp70 modulates silibinin-induced apoptosis in bladder cancer cells.** (A, B) Bladder cancer cell T24 were transfected with Hsp70 siRNA #1 before treatment with DMSO or silibinin (200 µM) for 24 hours, and stained with AnnexinV-FITC/PI, the apoptotic cells were analyzed by flow cytometry. (C) Hsp70-overexpressing plasmid was transfected into T24 cells, and then cells were incubated with or without 200 µM silibinin. Flow cytometry was used to detect the apoptotic cells. *P<0.05 vs control group or the indicated group. Results are shown as the mean ± standard deviation of three experimental repeats.

To verify the role of Hsp70 in silibinin-induced apoptosis, we knocked down Hsp70 in bladder cancer cell T24 using siRNAs and treated these transfected cells with DMSO or silibinin. Then the cells were stained with AnnexinV-FITC/PI and analyzed by flow cytometry. The results showed that Hsp70 knockdown enhanced silibinin-induced apoptosis (Fig. 3A and B). Meanwhile, when T24 cells were transfected with Hsp70-overexpressing plasmid, the apoptosis induced by silibinin was partially reversed (Fig. 3C). All in all, these results demonstrate that Hsp70 has an important role in silibinin-induced apoptosis in bladder cancer cells.

### Endogenous Hsp70 is involved in silibinin-induced apoptosis in bladder cancer cells

**Figure 4:**
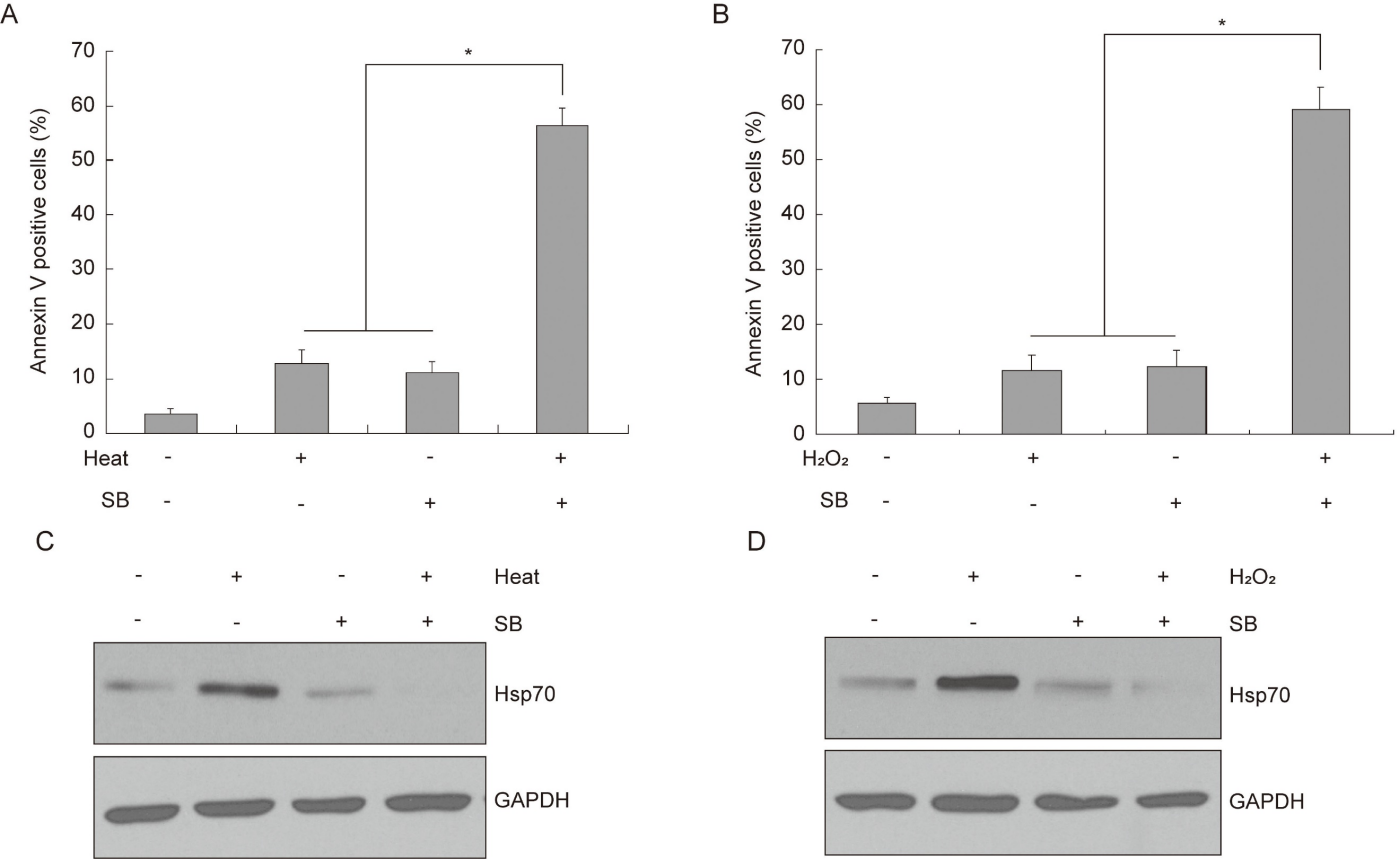
** Endogenous Hsp70 is involved in silibinin-induced apoptosis in bladder cancer cells.** (A, B) Bladder cancer cells T24 were exposed to 42 °C (A) or treated with 200 μM H_2_O_2_ for 24 hours (B), and then incubated with DMSO or silibinin (200 µM) for another 24 hours, the apoptotic cells were analyzed by flow cytometry. *P<0.05 vs the indicated group. Results are shown as the mean ± standard deviation of three experimental repeats. (C, D) The expression of Hsp70 was analyzed by western blotting.

Previous studies demonstrated that various stimuli, including hyperthermia and oxidative stress could induce overexpression of endogenous Hsp70, which can elicit protective responses to prevent cell death^23^, ^24^. Since we have demonstrated exogenous Hsp70 transfected into bladder cancer cells could inhibit silibinin-induced apoptosis, the underlying function of endogenous Hsp70 needs to be investigated. As shown in Figure 4A and B, silibinin treatment markedly increased the apoptotic cells under either heat or H_2_O_2_ treatment. Western blotting analysis showed that both heat and H_2_O_2_ treatment significantly induced the upregulation of endogenous Hsp70. Meanwhile, silibinin treatment decreased Hsp70 expression remarkably (Fig. 4C and D), suggesting a possible role of Hsp70 in silibinin-induced apoptosis in bladder cancer cells.

### Silibinin induces apoptosis in bladder cancer cells via inhibiting HSF1/Hsp70 pathway

**Figure 5:**
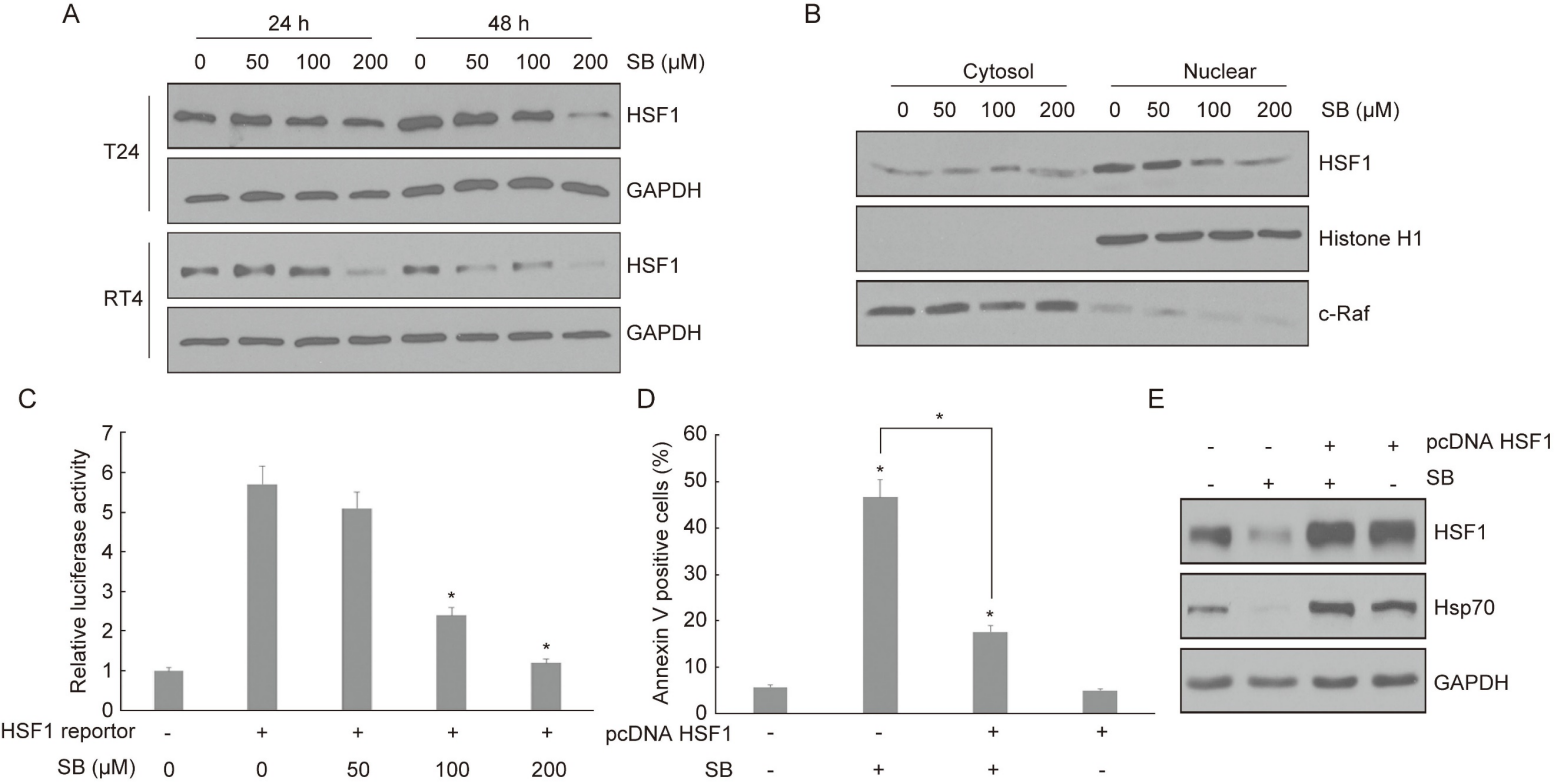
** Silibinin induces apoptosis in bladder cancer cells *via* inhibiting HSF1/ Hsp70 pathway.** Bladder cancer cells T24 and RT4 were treated with different concentrations of silibinin (0, 50, 100, 200 µM) for 24 or 48 hours, the expression of HSF1 was detected by western blotting. (B) The expression of HSF1 in cytosol and nuclear was analyzed using western blotting. (C) HSF1 reporter gene activity in silibinin treated bladder cancer cells was detected by dual-luciferase assay. (D, E) The bladder cancer cells were transfected with HSF1 plasmid, then the apoptosis rate was detected using flow cytometry (D) and the expressions of HSF1 and Hsp70 were detected by western blotting (E). *P<0.05 vs control group or the indicated group. Results are shown as the mean ± standard deviation of three experimental repeats.

To further detect the underlying mechanism of Hsp70 in silibinin-induced apoptosis, we focus on the key transcription factor HSF1. Bladder cancer cells T24 and RT4 were treated with silibinin for 24 hours and 48 hours, and the expression of HSF1 was detected using western blotting. The results showed that silibinin inhibited the expression of HSF1 in a dose-dependent manner (Fig. 5A). Furthermore, we found that silibinin mainly inhibited HSF1 expression in nuclear fractions (Fig. 5B). Luciferase assay showed that silibinin treatment inhibited HSF1 reporter gene activity in a dose-dependent manner (Fig. 5C). When bladder cancer cells were transfected with HSF1 plasmid, the apoptosis induced by silibinin was partially reversed (Fig. 5D). Next, western blotting analysis showed that silibinin suppressed both the expression of HSF1 and Hsp70, and the inhibitory effect of silibinin on Hsp70 was reversed by overexpressing HSF1 (Fig. 5E).

### Silibinin activates Hsp70-regulated mitochondrial apoptotic pathway

**Figure 6:**
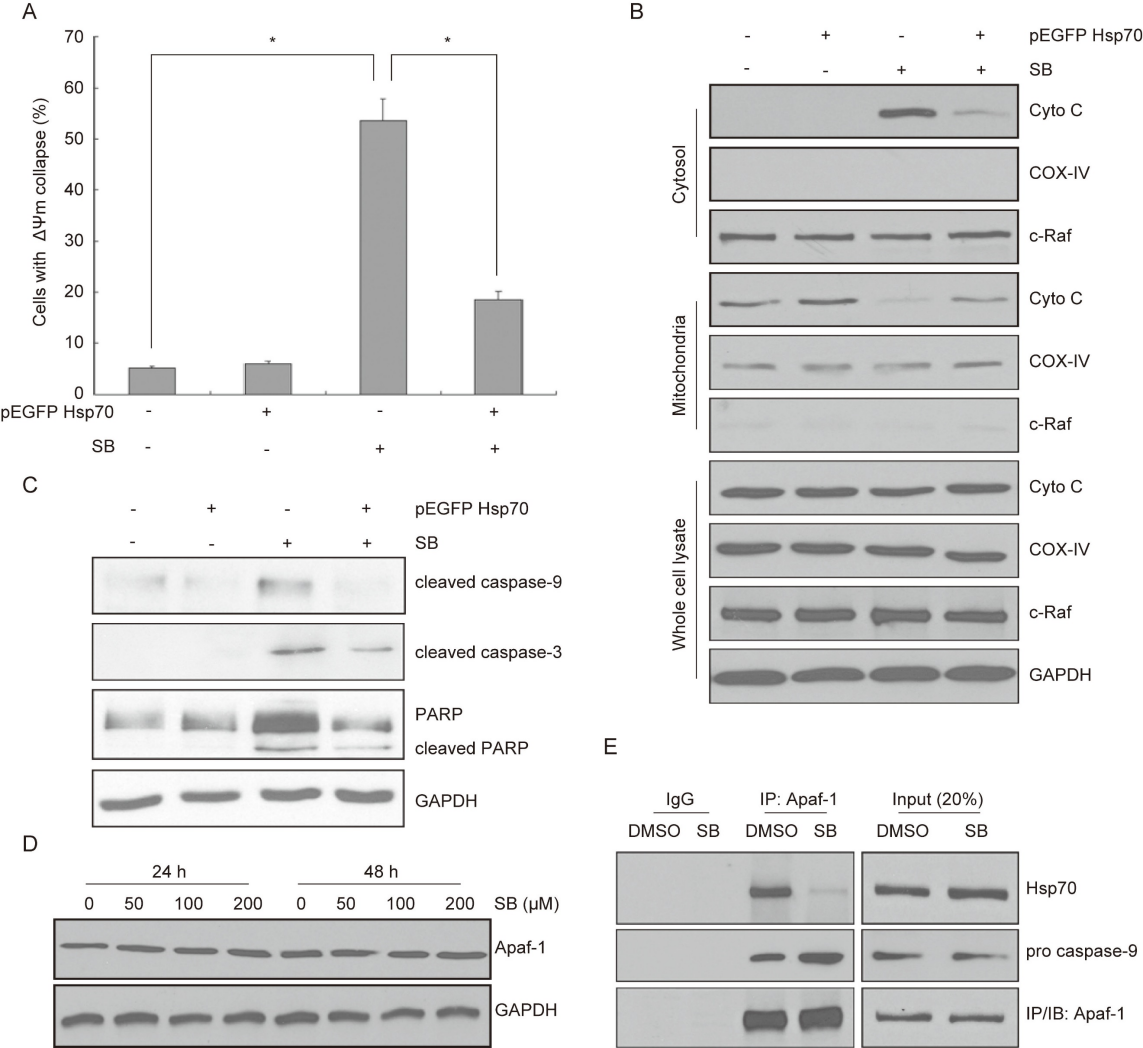
** Activation of Hsp70-regulated mitochondrial apoptotic pathway by silibinin.** (A) Bladder cancer cells T24 were transfected with Hsp70-overexpressing plasmid before treatment with DMSO or silibinin (200 µM) for 24 hours, and subjected to mitochondrial membrane potential (ΔΨm) assay, the ΔΨm were analyzed by flow cytometry. *P<0.05 vs the indicated group. Results are shown as the mean ± standard deviation of three experimental repeats. (B) Hsp70-overexpressing T24 cells were incubated with or without 200 µM silibinin. The expression of Cyto C in cytosol and mitochondria was analyzed using western blotting. (C) The expression of cleaved caspase-9, cleaved caspase-3 and PARP/cleaved PARP were detected by western blotting. (D) T24 cells was treated with different concentrations of silibinin (0, 50, 100, 200 µM) for 24 hours or 48 hours, and the expression of Apaf-1 was detected by western blotting. (E) Co-immunoprecipitation of endogenous Apaf-1, Hsp70 and pro caspase-9 were assayed in T24 cells following treatment with 200 µM silibinin.

To understand the potential mechanism related to Hsp70, we conducted mitochondrial membrane potential (ΔΨm) assay. Results showed that ΔΨm was increased by silibinin in bladder cancer cells, while this phenomenon could be partially reversed in Hsp70 overexpressed cells (Fig. 6A). Then we found that silibinin treatment increased cytosol cytochrome c (Cyto C) expression, while decreased its expression in mitochondria. Moreover, this effect was partially reversed by overexpressing Hsp70 (Fig. 6B). Western blotting analysis also showed that cleaved caspase-9, cleaved caspase-3 and cleaved PARP were activited by silibinin treatment, while partially reversed by overexpressing Hsp70 (Fig. 6C). Additionally, Hsp70 is known to bind to Apaf-1 and to prevent recruitment of caspase to the apoptosome complex for downstream caspases activation. Thus the relationship between Apaf-1 and Hsp70 was further investigated. The results showed that silibinin treatment had no effect on Apaf-1 expression (Fig. 6D). Co-immunoprecipitation assay showed that silibinin inhibited the interaction between Apaf-1 and Hsp70, thus increasing the recruitment of pro caspase-9 (Fig. 6E). In conclusion, Hsp70-regulated mitochondrial apoptotic pathway plays a key role in silibinin-induced apoptosis in bladder cancer cells.

### Anti-tumor effects of silibinin on bladder cancer cells *in vivo*

**Figure 7:**
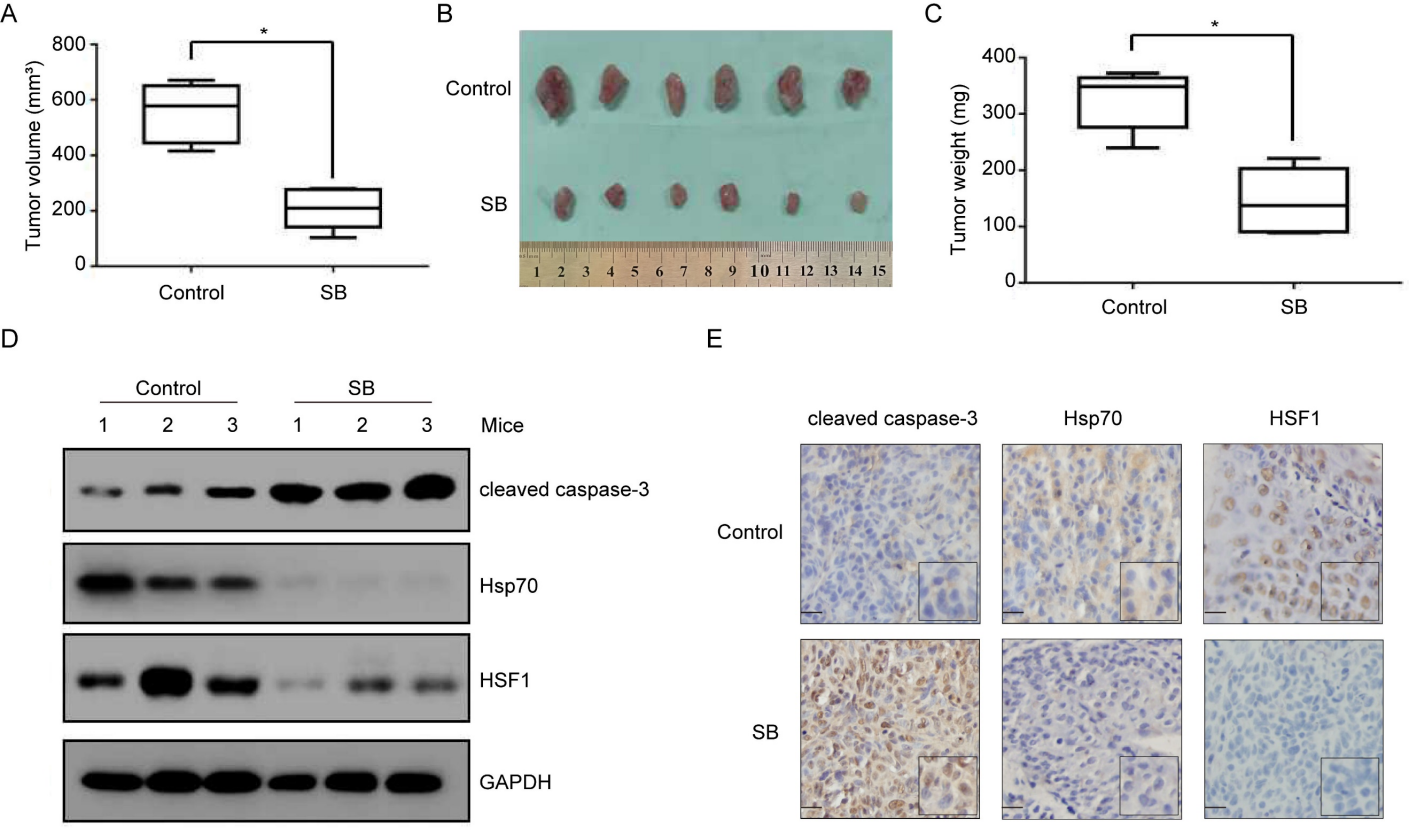
** Anti-tumor effects of silibinin on bladder cancer cells *in vivo*.** (A) T24 cells were subcutaneously injected into nude mice and intravenously treated with silibinin (100 mg/kg, three times per week). Statistical analysis of the tumor volumes which were measured every three days. *P<0.05 vs control group. (B) Subcutaneous xenograft tumors formed from different groups after treatment with silibinin for 30 days were dissected. (C) Statistical analysis of the weight of the dissected xenograft tumors. *P<0.05 vs control group. (D, E) The expression of cleaved caspase-3, Hsp70 and HSF1 in the tumors were measured by western blotting (D) and immunohistochemistry assay (E). Scale bar, 10 µm.

In order to validate whether silibinin treatment would inhibit bladder tumor growth *in vivo*, we established T24 xenograft tumor models in athymic nude mice. As shown in Figure 7A and B, tumors in T24 control group grew faster than those in silibinin-treated group *in vivo*. Consistently, silibinin treatment lessened T24 tumors weights (Fig. 7C). Western blotting (Fig. 7D) and immunohistochemical staining (Fig. 7E) results from tumor tissues demonstrated that silibinin suppressed bladder cancer growth through inhibiting HSF1/Hsp70 pathway.

## Discussion

Due to better patient tolerance and acceptance when compared to modern medicines, the plant-derived compounds have gained increasing attention and been applied in clinical for a long history. The utility of natural products as medicines on account of their biological properties including anti-cancer is still alive and well^25^. To date, numerous plant species have been screened for their medicinal use and plants especially those with ethnopharmacological uses particularly draws much attention. Among them, a flavonoids compounds extracted from milk thistle seeds called silibinin, has been shown to exert anti-cancer effects both *in vitro* and *in vivo*^7^-^12^. Though abundant evidences have verified its anti-cancer effects, few studies have explored its efficacy and underlying mechanism in bladder cancer. In the present study, we found that silibinin inhibits bladder cancer cells proliferation and induces apoptosis in concentration-dependent manner.

Heat shock proteins (HSPs) are a kind of highly conserved proteins that are widely expressed in all living organisms, possessing housekeeping functions^26^. Heat shock protein (HSP)70, which is closely related to the occurrence and development of cancer, as well as the resistance to apoptosis, is a member of the HSPs family and overexpressed in various cancers^27^. Recently, accumulating studies have demonstrated Hsp70 has strong cytoprotective properties in cancer, and is one of the most strongly and universally induced chaperones under pessimal stimulation^28^. Overexpressed Hsp70 prevents cell death triggered by various stimuli. On the contrary, depletion of Hsp70, either by antisense constructions or siRNA strategies, increases cell sensitivity to apoptotic stimuli^29^. In the current research, we discovered that silibinin exerted its anti-cancer function mainly through decreasing Hsp70 protein level. In eukaryotes, Hsp70 is regulated by a conserved family of heat shock transcription factors (HSFs). HSFs binds as a trimer to its cognate DNA motif, the heat shock element (HSE), in the promoters and enhancers of its target chaperone genes. The HSFs family consists of four members: HSF1, HSF2, HSF3, and HSF4. HSF1, HSF2, and HSF4 had been identified in mammals, while HSF3 was described in chicken. Researches have suggested that HSF1 is the master transcriptional regulator for heat shock proteins (HSPs) expression^30^. Our results found that silibinin inhibits Hsp70 through downregulating HSF1 level.

Hsp70 has been shown to inhibit the apoptotic pathways through different molecular mechanisms. In terms of pre-mitochondrial level, Hsp70 could inhibit JNK activation and caspase-3 activation in apoptosis via binding to JNK1^31^. At the mitochondrial level, Hsp70 prevents mitochondrial membrane permeabilization and release of Cyto C and AIF by inhibiting Bax translocation and insertion into the outer mitochondrial membrane^32^. When it comes to the post-mitochondrial level, Hsp70 has been demonstrated to bind directly to Apaf-1, thereby preventing the recruitment of pro caspase-9 to the apoptosome^33^. To verify whether silibinin-induced apoptosis through the mitochondrial pathway, we detected key molecules involved in this pathway and found that ΔΨm and cytosol Cyto C were increased by silibinin, while mitochondrial Cyto C was decreased, accompanied by an increase of cleaved caspase-9, cleaved caspase-3 and cleaved PARP. Moreover, we discovered that silibinin could increase the recruitment of pro caspase-9 by directly disturbing the interaction between Apaf-1 and Hsp70.

In summary, our study shows that silibinin inhibits cell proliferation and induces apoptosis in bladder cancer cells and Hsp70 plays an important role in silibinin-induced apoptosis. Mechanically, HSF1/Hsp70-regulated mitochondrial apoptotic pathway is involved in silibinin-induced apoptosis in bladder cancer. So far, many patients with advanced bladder cancer are not completely cured by surgery with standard therapies, and our study may thus be meaningful for silibinin as a potential therapeutic strategy.
